# The Prevalence of Metabolic Syndrome and Its Related Risk Complications among Koreans

**DOI:** 10.3390/nu11081755

**Published:** 2019-07-30

**Authors:** Seung-Hoo Lee, Shuting Tao, Hak-Seon Kim

**Affiliations:** School of Hospitality & Tourism Management, Kyungsung University, 309 Suyoungro, Nam-Gu, Busan 48434, Korea

**Keywords:** metabolic syndrome, prevalence, risk complications, Korean National Health and Nutrition Survey, tuberculosis

## Abstract

There is an increasing number of metabolic syndrome (MetS) patients worldwide, and there is no exception in South Korea. The risk complications of metabolic syndrome have been investigated by many previous research studies, while no data on any current trends of MetS are available. Therefore, the present study investigates the recent prevalence of MetS and its associated risk complications in Korean adults by using the Korean National Health and Nutrition Examination Survey (KNHANES). The Survey respondents (*n* = 4744) were adults over the age of 30, and they had participated in KNHANES 2016, which is a health survey of a national representative sample of non-institutionalized civilian South Koreans. The cross-tabulation analysis was applied to figure out the general characteristics impacting on the prevalence of MetS; furthermore, the odds ratios and 95% confidence intervals (CIs) using multivariate logistic regression analysis were presented for the risk complications of MetS. Findings from this study indicated that subjective health status, family structure, age, income level, use of nutrition labelling and gender showed significant connections with the prevalence of MetS. The risk diseases, stroke (OR = 2.174, 95% CI = 1.377–3.433, *p* < 0.01), myocardial infarction (MI) (OR = 2.667, 95% CI = 1.474–4.824, *p* < 0.01) and diabetes (OR = 6.533, 95% CI = 4.963, *p* < 0.001) were explored and verified attributable to the prevalence of MetS. The findings in this study suggest that sociodemographic characteristics-concentrated strategies are vital to prevent the prevalence of MetS in South Korea, and relative risk complications ought to be cautiously dealt with as well.

## 1. Introduction

The metabolic syndrome (MetS) which is a combination of risk factors that consists of several correlations of metabolic origin, has been receiving more attention in recent years [1,2]. The 10 major causes of death in South Korea were cancer, heart disease, cerebrovascular disease, suicide, pneumonia, diabetes mellitus, chronic lower respiratory disease, liver disease, traffic/transit accidents and hypertensive diseases [3]. Among these causes, the non-communicable diseases such as comprising cancers, cardiovascular disease (CVD), diabetes, and chronic lung diseases, were preventable by recognizing the altering behavioral or intermediate risk factors like hypertension, pre-diabetic status, obesity and MetS [4]. MetS is reported as an important etiologic factor in the development of certain types of cancers [5], and the economic cost of cancer treatment has been steadily increasing. In 2012, the cause of 18,070 cancer patients in South Korea was found to be metabolic syndrome. The economic burden for cancer treatment was USD 199.8 million, and the direct and indirect costs were USD 124.5 million and USD 75.3 million, respectively [6].

That is, the trend of rising cost has been observed in Asian countries, especially in South Korea, posing a major challenge to public health professionals, as well as becoming a social and economic problem in present and in the near future [7,8].

Since Reaven first explained MetS in 1983, several researchers have attempted to define the concept of Mets [7,9,10]. Furthermore, the World Health Organization (WHO) defined the metabolic abnormality known as metabolic syndrome, and numerous studies have been released on the different criteria of metabolic syndrome [11]. MetS represents a major public health burden as defined by its prevalence, risk and etiologic fraction. Moreover, the impact of MetS is likely to increase because of the rapid development of the obesity epidemic [8]. MetS is a constellation of interrelated metabolic risk factors, and its syndrome is not a discrete entity known to be caused by a single factor. MetS shows significant differences in the components among different individuals. This variation works even greater among different races and ethnic groups [1]. That is to say, different variables are identified with the prevalence of metabolic syndrome, like country, region, gender, race and time, as well. Accordingly, the corresponding health care for the public needs to be conducted based on these differences. Likewise, the study of Grundy et al., suggests that lifestyle interventions are the initial therapies recommended for the treatment of MetS [1].

In South Korea, there were a great number of studies associated with MetS conducted with KNHANES. Park et al. performed the research which indicated that higher body mass index (BMI) and current smoking were identified as independent, modifiable risk factors for the prevalence of MetS [12]; Oh and Kim used KNHANES for revealing that rural residents showed significantly lower likelihood of having metabolic syndrome compared with urban residents [13]. Kim indicated that groups of health living conditions such as dietary pattern, body image, muscle mass, and fat mass were differentially associated with metabolic syndrome risk factors [14]. Tran et al. applied the data of KNHANES from 2008 to 2013, and the findings of their study demonstrated that age and obesity were linked with increased MetS in both men and women. Meanwhile, smoking and alcohol drinking were significantly related to MetS in men [4]. Like aforementioned, the trend of the prevalence of MetS differs from time, and it is necessary and instrumental for us to grasp the lasting trend of MetS to develop effective strategies for the prevention of MetS. In addition, variables affecting on MetS and its risk complications are pivotal to be characterized and synthesized. Therefore, the findings of this study can provide basic data for controlling the prevalence of MetS in South Korea, with appropriate MetS prevention and health-related policies for Korea.

## 2. Subjects and Methods

### 2.1. Data Source

The database was from The Korean National Health and Nutrition Survey (KNHANES) 2016, which was conducted by the Korean Ministry of Health and Welfare. In brief, KNHANES is an annual surveillance system that collects information about participants’ demographic, social, health and nutritional status through the following components surveys using representative country samples: A health interview survey, a health behaviors survey, a health examination survey and a nutrition survey [13,14]. To sum up, KNHANES is a nationwide cross-sectional survey, and its target population comprises nationally representative non-institutionalized civilians in South Korea.

KNHANES data are valuable sources to monitor how risk factors and diseases are changing, and it is also important to identify target groups in need of interventions. For the health interview and examination, nurses and relative staff were trained to carry out anthropometric measurements, serum collection, blood pressure measurements and questionnaires management. A general questionnaire was administered to estimate the basic demographic, socioeconomic, dietary and medical history details of each respondent; for instance: Region, gender, age, their level of education, income and marital status [13,14]. Dietary measurement, height and weight were obtained through standardized techniques and calibrated equipment. Waist circumference (WC) was measured as the narrowest point from the lower border of the rib cage to the iliac crest. 

The participants measured their height and weight, wearing light clothes without shoes, and then the calculation of the body mass index (BMI) was performed by dividing the weight of the participant by the square of his/her height (kg/m^2^). Blood pressure was measured while the participants were seated, by the use of a sphygmomanometer. High-speed glucose, fasting insulin, total cholesterol, triglycerides (TG), and HDL were analyzed in a central certification laboratory.

The assessment of nutrition intake was conducted in such a way that the usual dietary habits were ascertained by the 24-h recall method before assessing dietary intake, and nutrient intake was estimated with the Can-Pro 2.0 nutrient intake assessment software developed by the Korean Nutrition Society [15]. As for the information of dietary intake, it was aggregated through a face-to-face interview using a validated food-frequency semi-questionnaire questionnaire for Koreans, and 63 food items of the participants’ consumption were considered [4,15].

### 2.2. Research Subjects

A total of 8150 people participated in the survey. Finally, a total of 4,744 men and women over 30 years old (58.21%) were included in the data analysis. In South Korea, most studies have demonstrated that the incidence of metabolic syndrome (MetS) is significantly higher over 30 years of age [16]. Therefore, this study performed the research, concentrating on participants of 30 years or over. These participants are divided into two groups, with the normal group and the MetS group. The diagnostic criteria of MetS have been modified several times, and it is also applied differently depending upon the region [5]. Currently, in Korea, the definition of MetS is composed by the modified Third National Cholesterol Education Program Expert Panel on Detection, Evaluation, and Treatment of High Blood Cholesterol in Adults (NCEP-ATP III) and the specific values for WC provided by the World Health Organization and the Korean Society for the Study of Obesity. This study defined subjects as having metabolic syndrome who had met three or more of the following criteria: (1) Abdominal obesity (the Asia-Pacific criteria for obesity based on WC: Men ≥ 90 cm and women ≥ 85 cm); (2) triglycerides ≥ 150 mg/dL; (3) fasting glucose levels ≥ 100 mg/dL; (4) high-density lipoprotein (HDL) cholesterol < 40mg/dL in men or < 50 mg/dL in women; (5) hypertension as blood pressure ≥ 130/85 mmHg [11,13,17].

In this study, several sociodemographic characteristics were included. The division of the area is the metropolitan city covered by Seoul, Gyeonggi, Busan, Daegu, Incheon, Gwangju, Daejeon and Ulsan, while other administrative districts were defined as others. The family structure applied in this study has two categories, single generation and multi-generation. The single generation represented a childless family or single persons which hold only one generation, while the multi-generation represented the nuclear family, stepfamily, extended family, etc., that included more than one generation in the family. In the report of KNHANES 2016, the income level of participants were clustered into four levels: Low, middle low, middle high and high. Low and middle low were defined as low, while middle high and high were defined as high in this study.

### 2.3. Statistical Analysis

The statistical analysis in this study was performed by SPSS 25.0 for Windows (SPSS Inc., Chicago, IL, USA). The relationship between the general characteristics of respondents from two groups (normal and metabolic syndrome) was assessed using the Chi-square test, and the results were presented as Means ± SE. In addition, the generalized linear model was applied to compare numerical variables such as anthropometric measurements, metabolic risk factors and nutrient intake by normal subjects and MetS subjects [13,14]. The multivariate logistic regression analysis was applied to estimate the predictors affecting the increase in the prevalence of MetS. Odds ratios (ORs) with a 95% confidence interval (95% CI) were calculated for the analysis of the association between the two groups and their risk factors for MetS. A *p* value of less than 0.05 was considered statistically significant.

## 3. Results

The general sociodemographic characteristics of the study population, stratified by two groups (normal and MetS group), were given in Table 1. There was no significant difference for the frequency of dining out (*p* = 0.213) and area (*p* = 0.190) between the normal group and the MetS group. Variables with subjective health status, family structure, age and gender demonstrated significance with *p* < 0.001; in addition, the income level and use of nutrition labeling were also significantly different, with *p* < 0.05 and *p* < 0.01. In terms of subjective health status, when participants held the opinion that they have the bad health status, the distribution between normal group and MetS group is 55.3% vs. 44.7%, and the proportional share of the MetS group was decreasing with an increment of the positive subjective health status. This result showed that participants had, to a great extent, a clear judgment of their health status. The prevalence of MetS in multi-generation was lower than a single generation with a proportional share of 35.2% to 41.8%, respectively. As for the age, the distribution of normal and MetS group is obviously different in the age range of 30–39 years, and with the age increasing, the difference is getting smaller—the prevalence of MetS of 70 years or over was almost equally distributed (53.8% in the normal group vs. 46.2% in MetS group). The higher the income level with the prevalence of Mets is 34.8%, while the prevalence of MetS in low-income level is 38.6%, and this finding verified that the better the socioeconomic position of an individual, the lower is the risk of premature coronary heart disease, which also means the lower incidence of MetS [18,19,20]. Use of nutrition labeling (*p* < 0.01) also significantly impacted upon the prevalence of MetS. The prevalence of MetS for the use of nutrition labeling (28.3%) is lower than no use of nutrition labeling (35.9%). The distributive differences between gender were significant with *p* < 0.001 (prevalence of MetS in male 44.4% vs. female 37.1%), and women had a 7.3% higher prevalence than men did. This result is consistent with many other previous studies conducted in different areas [21,22].

Table 2. revealed the ORs and adjusted ORs (95% confidence interval (CI)) for associations between sociodemographic variables (which were obtained from the previous analysis shown in Table 1) and the MetS group.

For the prevalence of MetS, the impact of demographic variables, especially the family structure (single generation), was significant (1.129–1.605, *p* < 0.001) and the gender (female) as well (1.409–1.867, *p* < 0.001).

The comparison of related nutrition factors for normal and MetS groups is presented in Table 3. Systolic blood pressure of the MetS group was significantly higher than the normal group (127.21 ± 0.485 vs. 114.72 ± 0.556, *p* < 0.001) and the same as fasting blood glucose (113.889 ± 0.931 vs. 95.05 ± 0.950, *p* < 0.001), triglyceride (225.47 ± 5.411 vs. 105.988 ± 2.082, *p* < 0.001), weight (71.06 ± 0.379 vs. 61.71 ± 0.463, *p* < 0.001) and WC (89.57 ± 0.268 vs. 80.39 ± 0.301, *p* < 0.001). However, the HDL-cholesterol of the MetS group was significantly lower than the normal group (42.68 ± 0.305 vs. 55.176 ± 0.422, *p* < 0.001). Diastolic blood pressure and BMI were slightly higher in the MetS group. The result of this section, to a great extent, verifies the diagnostic criteria of MetS. The subtle difference of BMI and WC, and the index of obesity (*p* < 0.01), which shows the normal group is slightly higher than the MetS group, respectively, corresponded with the change of the MetS diagnostic criteria, from obesity to WC, which is more rational and scientific [23,24,25]. Saturated fatty acid (SFA) (*p* = 0.239) did not impact upon the MetS significantly.

The difference of the odds ratios (95% CI) for MetS risk factors between normal and MetS groups was calculated in Table 4. All of the following risk factors were indicated to be significantly associated with MetS (*p* < 0.001). The results also confirmed the diagnostic criteria of MetS, as the results of this study showed that abdominal obesity (waist circumference), triglycerides, fasting glucose levels, HDL cholesterol and hypertension (systolic blood pressure and diastolic blood pressure) were significant with a *p* value < 0.001.

Table 5 presented the odds ratios (95% CI) for chronic diseases associations between normal and MetS groups. The stroke (1.318–3.495, *p* < 0.01), myocardial infarction (MI) (1.301–4.810, *p* < 0.01) and diabetes values (3.592–5.953, *p* < 0.001) were significantly associated with the MetS group, which to a great extent, showed that these three chronic diseases are greatly potential risk factors for MetS. Meanwhile, the study by Boden-Albala et al. showed that there was a significant association between MetS and ischemic stroke risk, regardless of age, education, physical activity, alcohol use and other confounding factors, including current smoking (a study from northern Manhattan study) [8]. The tuberculosis (TB) was not significantly associated with MetS, with *p* = 0.081. Prior study suggest that TB may increase the risk of developing diabetes, which is significantly related to MetS [26].

## 4. Discussion

The results of this study reveal the current circumstance of MetS in South Korea based on the data from KNHANES 2016; additionally, the differences between normal and MetS groups of middle-age (30 years or over) Koreans’ metabolic syndrome abnormality and related factors include sociodemographic factors, nutritional factors and diseases relative to the prevalence of MetS were examined and testified to as well. The results of this study showed that subjective health status recognition, family structure, age, income level, use of nutrition labeling and gender were greatly associated with MetS. In particular, when the recognition of subjective health status is “bad”, the distribution of participants in MetS and the normal group is 44.7% vs. 55.3% which implied that in this segment, the MetS group does not possess a good recognition of their health status. Furthermore, the prevalence of MetS in males is higher than in females with the 44.4% male in the MetS group while there is 37.1% female in the MetS group. The previous study also confirmed that men have a higher risk factor level compared with women [12,27], which suggested that in the future, MetS-preventive treatments should be sex-specific. The findings in this study suggest that the difference lies in some sociodemographic characteristics and nutrient factors, providing possible explanations of the difference in normal and MetS groups. To sum up, the prevalence of MetS was not significantly associated with the geographical location of participants, while the social and cultural facets of the respondents more affected the prevalence of MetS [9,27].

The risk complications of the prevalence of MetS were demonstrated, consisting of stroke, MI, tuberculosis and diabetes. Likewise, the study conducted by John et al. presents that MetS is significantly associated with self-reported MI, stroke and MI/stroke, which to a great extent showed that MetS has a clinical utility in identifying patients at increased risk of MI and stroke [28], though the data source of this study is the Third National Health and Nutrition Examination Survey in the US, and the value to evaluate the association between MI, stroke and MetS still exists.

Though the study is finished, the limitation still exists. Thus, the results of this study should be interpreted with caution in the light of these limitations. First, as many research studies have mentioned previously, assessing the dietary variables based on a single day 24-h diet recall may lead to the deviation of the day-to-day usual nutrient intake [29,30,31]. Second, the risk components of the prevalence of MetS are changeable varying the development of socio-economy, the alteration of dietary habits and sleep duration [32,33]; so do the diagnostic criteria of MetS. Therefore, with different time and social background, the offset of the diagnosis exists.

## 5. Conclusions

Findings of this study contribute to the knowledge about the current prevalence trend of MetS in South Korea and the relative risk factors of MetS, and more importantly, the risk complications of MetS in South Korea were performed and verified as well. In South Korea, the Korean government and the Korean National Assembly approved laws on health promotion and disease prevention, and one of the main targets is to reduce smoking, drinking alcohol and obesity in Health Plan 2020. This policy includes lifestyle interventions and public education about healthy eating behaviors with physical activity [2]. Fortunately, nowadays, there is a decreasing prevalence of abdominal obesity and smoking in South Korea [4]. Understanding the KNHAES data, a greater awareness of MetS and its positive outcomes will be helpful for the prevention of MetS and the treatment of health risk factors among Korean adults [34]. Multidimensional approaches are needed to be conducted to prevent the future prevalence of MetS.

## Figures and Tables

**Table 1:** General sociodemographic characteristics of the normal group and the metabolic syndrome (MetS) group.

Variables		30 Years or Over (*n* = 4744)			
		Normal Group (*n* = 2993)	MetS Group (*n* = 1751)	Chi-Square	*p*
Frequency of dining out	Low (<5 times a week)	63.9%	36.1%	2.739	0.213
	High (>5 times a week)	61.4%	38.6%		
Area	Metropolitan cities	64.2%	35.8%	3.134	0.190
	Others	61.6%	38.4%		
Subjective health status	Bad	55.3%	44.7%	45.900	<0.001
	General	62.6%	37.4%		
	Good	69.9%	30.1%		
Family structure	Single generation	58.2%	41.8%	17.282	<0.001
	Multi-generation	64.8%	35.2%		
Age (y)	30–39 y	76.3%	23.7%	132.290	<0.001
	40–49 y	64.9%	35.1%		
	50–59 y	58.9%	41.1%		
	60–69 y	53.3%	46.7%		
	≥70 y	53.8%	46.2%		
Income level	Low	61.4%	38.6%	7.350	0.041
	High	65.2%	34.8%		
Use of nutrition labelling	No	64.1%	35.9%	12.246	0.006
	Yes	71.7%	28.3%		
Gender	Male	55.6%	44.4%	97.015	<0.001
	Female	62.9%	37.1%		

**Table 2:** The odds ratios (95% CI) for demographic variables according to normal and MetS groups.

Variables	Normal Group (*n* = 2993)	MetS Group (*n* = 1751)		
	OR	OR	95% CI	*p*
Use of nutrition labelling	1 (Yes)	1.403	1.099–1.733	0.012
Gender	1 (Female)	1.631	1.409–1.867	<0.001
Income level	1 (High)	1.238	1.077–1.433	<0.001
Family structure	1 (Single generation)	1.346	1.129–1.605	<0.001

**Table 3:** Comparison between normal and MetS groups of associated nutrition factors.

	30 Years or Over (*n* = 4744)				
	Normal Group (*n* = 2993)		MetS Group (*n* = 1751)		*p*
	Mean	SE	Mean	SE	
Protein (g)	70.84	1.623	70.82	1.408	0.990
Energy (kcal)	1991.72	37.556	2046.72	33.090	0.144
Obesity (body mass index (BMI))	45.48	1.345	43.75	1.182	0.198
Saturated fatty acid (g/d)	13.09	0.432	12.58	0.374	0.239
Monounsaturated fatty acid (g/d)	14.66	0.490	14.04	0.434	0.210
Cholesterol (mg/dL)	275.28	10.848	259.91	9.917	0.158
Carbohydrate (g/dL)	298.948	4.703	301.14	4.293	0.641
Dietary fiber (g/dL)	23.53	0.502	23.80	0.457	0.591
Calcium (mg/dL)	474.30	12.341	475.18	10.736	0.944
Silver (mg)	1061.00	20.913	1061.37	18.742	0.986
Iron (mg)	17.23	0.487	17.30	0.373	0.885
Salt (mg)	3805.86	101.680	3939.20	90.650	0.191
Vitamin A (mg/dL)	710.469	31.033	695.93	26.647	0.640
Carotene (mg/dL)	3413.08	128.756	3360.20	109.170	0.682
Retinal (mg/dL)	122.57	17.392	110.623	14.202	0.493
Thiamine (mg/dL)	1.97	0.043	2.01	0.038	0.398
Vitamin C (mg/dL)	111.09	4.311	104.99	3.850	0.138
Systolic blood pressure (mmHg)	114.72	0.556	127.21	0.485	<0.001
Diastolic blood pressure (mmHg)	74.376	0.367	80.67	0.315	<0.001
Weight (kg)	61.71	0.463	71.06	0.379	<0.001
Waist circumference (cm)	80.39	0.301	89.57	0.268	<0.001
BMI (kg/m^2^)	23.05	0.118	26.24	0.099	<0.001
Fasting blood glucose (mg/dL)	95.05	0.950	113.889	0.931	<0.001
Triglyceride (mg/dL)	105.988	2.082	225.47	5.411	<0.001
high-density lipoprotein (HDL) -cholesterol (mg/dL)	55.176	0.422	42.68	0.305	<0.001

**Table 4:** Odds ratios (95% CI) for MetS risk factors between the normal group and the MetS group.

	Normal Group (*n* = 2993)	MetS Group (*n* = 1751)		*p*
	OR	OR	95% CI	
BMI (1 unit increase)	1	1.360	1.268–1.459	<0.001
Fasting blood glucose (10 units)	1	1.613	1.416–1.838	<0.001
Triglyceride (10 units)	1	1.130	1.092–1.170	<0.001
HDL-cholesterol (1 unit)	1	0.901	0.887–0.915	<0.001
Systolic blood pressure (1 unit)	1	1.057	1.047–1.066	<0.001
Diastolic blood pressure (1 unit)	1	1.016	1.001–1.030	<0.001
Waist circumference (1 unit)	1	1.123	1.110–1.135	<0.001

**Table 5:** Odds ratios (95% CI) for chronic diseases associations between normal and MetS groups.

	Normal Group (*n* = 2993)	MetS Group (*n* = 1751)		*p*
	OR	OR	95% CI	
Stroke	1	2.146	1.318–3.495	0.004
Myocardial infarction	1	2.321	1.301–4.810	0.004
Tuberculosis	1	0.703	0.474–1.045	0.081
Diabetes	1	4.624	3.592–5.953	<0.001

## References

[1] Grundy S.M., Cleeman J.I., Daniel S.R., Donato K.A., Eckel R.H., Franklin B.A., Spertus J.A. (2005). Diagnosis and management of the metabolic syndrome: An American Heart Association/National Heart, Lung, and Blood Institute scientific statement. Circulation.

[2] Grundy S.M. (2008). Metabolic syndrome pandemic. Arterioscler. Thromb. Vasc. Biol..

[3] Shin H.Y., Lee J.Y., Song J., Lee S., Lee J., Lim B., Huh S. (2015). Cause-of-death statistics in the Republic of Korea, 2014. J. Korean Med. Assoc..

[4] Tran B.T., Jeong B.Y., Oh J.K. (2017). The prevalence trend of metabolic syndrome and its components and risk factors in Korean adults: Results from the Korean National Health and Nutrition Examination Survey 2008–2013. BMC Public Health.

[5] Braun S., Bitton-Worms K., LeRoith D. (2011). The link between the metabolic syndrome and cancer. J. Biol. Sci..

[6] Kim D., Yoon S.J., Gong Y.H., Kim Y.A., Seo H.Y., Yoon J., Kim A.R. (2015). The economic burden of cancers attributable to metabolic syndrome in Korea. J. Prev. Med. Public Health.

[7] Lim S., Shin H., Song J.H., Kwak S.H., Kang S.M., Yoon J.W., Jang H.C. (2011). Increasing prevalence of metabolic syndrome in Korea: The Korean National Health and Nutrition Examination Survey for 1998–2007. Diabetes Care.

[8] Nstel P., Lyu R., Low L.P. (2007). Metabolic syndrome: Recent prevalence in East and Southeast Asian populations. Asia Pac. J. Clin. Nutr..

[9] Haffner S., Taegtmeyer H. (2003). Epidemic obesity and the metabolic syndrome. Circulation.

[10] Boden-Albala B., Sacco R.L., Lee H.S., Grahame-Clarke C., Rundek T., Elkind M.V., Paik M.C. (2008). Metabolic syndrome and ischemic stroke risk: Northern Manhattan Study. Stroke.

[11] World Health Organisation (1999). Definition, Diagnosis and Classification of Diabetes Mellitus and Its Complications. Part 1: Diagnosis and Classification of Diabetes Mellitus.

[12] Park H.S., Oh S.W., Cho S.I., Choi W.H., Kim Y.S. (2004). The metabolic syndrome and associated lifestyle factors among South Korean adults. J. Epidemiol..

[13] Oh C., Kim H.S. (2016). Metabolic syndrome and its related factors among Korean elderly in urban and rural Areas. Culin. Sci. Hosp. Res..

[14] Kim H.S. (2014). Metabolic syndrome related health inequalities in Korean elderly: Korean National Health and Nutrition Examination Survey (KNHAES). Int. J. Equity Health.

[15] Kim D.W., Song S., Lee J.E., Oh K., Shim J., Kweon S., Joung H. (2015). Reproducibility and validity of an FFQ developed for the Korea National Health and Nutrition Examination Survey (KNHANES). Public Health Nutr..

[16] Lee S.Y., Park H.S., Kim D.J., Han J.H., Kim S.M., Cho G.J., Oh S.J. (2007). Appropriate waist circumference cutoff points for central obesity in Korean adults. Diabetes Res. Clin. Pract..

[17] Enkhmaa B., Shiwaku K., Anuurad E., Nogi A., Kitajima K., Yamaski M., Yamane Y. (2005). Prevalence of the metabolic syndrome using the Third Report of the National Cholesterol Educational Program Expert Panel on Detection, Evaluation, and Treatment of High Blood Cholesterol in Adults (ATP III) and the modified ATP III definitions for Japanese and Mongolians. Clin. Chim. Acta.

[18] Kong H.J., Kim J., Hwang E.J., Hong J.Y., Kin S.H. (2014). Effects of healthcare smart home exercise program on the metabolic syndrome risk factors of obese elderly women. J. Korean Gerontol. Soc..

[19] Kim S., So W.Y. (2016). Prevalence of metabolic syndrome among Korean adolescents according to the national cholesterol education program, adult treatment panel iii and international diabetes federation. Nutrients.

[20] Ranasinghe P., Mathangasinghe Y., Jayawardena R., Hills A.P., Misra A. (2017). Prevalence and trends of metabolic syndrome among adults in the asia-pacific region: A systematic review. BMC Public Health.

[21] Lakka H.M., Laaksonen D.E., Lakka T.A., Niskanen L.K., Kumpusalo E., Tuomilehto J., Salonen J.T. (2002). The metabolic syndrome and total and cardiovascular disease mortality in middle-aged men. JAMA.

[22] Alberti K.G. (2005). IDF epidemiology task force consensus group: The metabolic syndrome: A new worldwide definition. Lancet.

[23] Alberti K.G.M.M., Zimmet P.Z. (1998). Definition, diagnosis and classification of diabetes mellitus and its complications. Part 1: Diagnosis and classification of diabetes mellitus. Provisional report of a WHO consultation. Diabet. Med..

[24] Ford E.S., Giles W.H., Dietz W.H. (2002). Prevalence of the metabolic syndrome among US adults: Findings from the third National Health and Nutrition Examination Survey. JAMA.

[25] Oh C., Kim H.S., No J.K. (2015). Impact of dining out on nutritional intake and metabolic syndrome risk factors: Data from the 2011 Korean National Health and Nutrition Examination Survey. Br. J. Nutr..

[26] Young F., Critchley J.A., Johnstone L.K., Unwin N.C. (2009). A review of co-morbidity between infectious and chronic disease in Sub Saharan Africa: TB and diabetes mellitus, HIV and metabolic syndrome, and the impact of globalization. Glob. Health.

[27] Van Dijk S.J., Feskens E.J., Bos M.B., Hoelen D.W., Heijligenberg R., Bromhaar M.G., Afman L.A. (2009). A saturated fatty acid–rich diet induces an obesity-linked proinflammatory gene expression profile in adipose tissue of subjects at risk of metabolic syndrome. Am. J. Clin. Nutr..

[28] Brunner E.J., Marmot M.G., Nanchahal K., Shipley M.J., Stansfeld S.A., Juneja M., Alberti K.G.M. (1997). Social inequality in coronary risk: Central obesity and the metabolic syndrome. Evidence from the Whitehall II study. Diabetologia.

[29] Du Y., Oh C., No J. (2018). Associations between sarcopenia and metabolic risk factors: A systematic review and meta-analysis. J. Obes. Metab. Syndr..

[30] Ninomiya J.K., L’Italien G., Criqui M.H., Whyte J.L., Gamst A., Chen R.S. (2004). Association of the metabolic syndrome with history of myocardial infarction and stroke in the Third National Health and Nutrition Examination Survey. Circulation.

[31] Kim H.S., Oh C., No J.K. (2016). Can nutrition label recognition or usage affect nutrition intake according to age?. Nutrition.

[32] Um Y.J., Oh S.W., Lee C.M., Kwon H.T., Joh H.K., Kim Y.J., Ahn S.H. (2015). Dietary fat intake and the risk of metabolic syndrome in Korean adults. J. Fam. Med..

[33] Choi K.M., Lee J.S., Park H.S., Baik S.H., Choi D.S., Kim S.M. (2008). Relationship between sleep duration and the metabolic syndrome: Korean National Health and Nutrition Survey 2001. Int. J. Obes..

[34] Oh C., Kim H.S. (2018). Understanding of nutrition labelling use and related factors among Korean adults. Culin. Sci. Hosp. Res..

